# Implementation of critical care staff based rapid response team. effect of rapid response system to the unpredicted death

**DOI:** 10.1186/2197-425X-3-S1-A67

**Published:** 2015-10-01

**Authors:** M Arai, T Koike, M Moriyasu, S Ito, T Ootsuka, T Inagaki, J Hattori, K Yoshino, M Kuroiwa

**Affiliations:** School of Medicine, Division of Intensive Care Medicine, Department of Research and Development Center for New Medical Frontiers, Kitasato University, Sagamihara, Japan; Division of Respiratory Care and Rapid Response System, Kitasato University Hospital, Sagamihara, Japan; School of Medicine, Department of Anesthesiology, Kitasato University, Sagamihara, Japan; School of Medicine, Department of Emeregency and Critical Care Medicine, Kitasato University, Sagamihara, Japan

## Introduction

Our hospital is a tertiary 1000-bed hospital. In 2011, rapid response system (RRS) was introduced in the hospital. Our rapid response team (RRT) includes critical care physicians, critical care nurses and physical therapists. Usually, team members are working at ICU. They also provides respiratory care or critical care interventions to the patients in the wards, after discharged from ICU. It is just like a Critical Care Outreach Team (CCOT), however, we call the system, Respiratory Support Team (RST) in our country. Once the RRS is activated, the team immediately responds to any type of call during 24 hours a day. The RRS is activated according to predefined criteria.

## Objectives

The object of the study is to make descriptive evaluation of our RRT activity. The impact of RRS on unpredicted hospital death (UHD) is also evaluated.

## Methods

The activity of RRT was collected prospectively in a database file. The reason for RRS activation, Modified Early Warning Score (MEWS) at the time of RRS activation, intervention at scene was evaluated from the database. The medical records of the patients who admitted to the hospital from March 2009 to March 2014 were collected retrospectively. To examine the efficacy of RRS, the number of UHD in Pre RRS period and Post RRS period were compared. UHD was defined as the case which the patients were attempted to resuscitate during cardiac arrest. Resuscitation related terms in the medical records were investigated and it was considered as an evidence of resuscitation. Statistical comparisons were made by Mann-Whitney's U-test for un-paried data (MESW and UHD).

## Results

The number of RRS activation was 325 during 4 years. 69% of calls were made by ward nurses. The reason for RRS activation was, SpO2 < 92% (85%), worried to the patient (60%), dyspnea (50%), respiratory rate>25/min (22%), noisy airway (22%), loss of consciousness (12%), tachycardia>120/min (11%). In the early stage of RRS implementation, the average of MEWS was 5.82 ± 2.44, however at the end of 2013, MEWS was 3.77 ± 2.21 (p < 0.001). Most frequent intervention at the scene was oxygen administration (60%), suction of the airway (54%), manual ventilation (27%), endotracheal intubation (21%), start of non-invasive positive pressure ventilation (20%). 35% of the cases were admitted to ICU. The total number of the patients who admitted to the hospital during 5 years was 109557 (Pre 50991, Post 58596). UHD in each phase was Pre RRS 0.58 ± 0.54 vs Post RRS 0.30 ± 0.4 (per 1000 admission per month) (p = 0.019).

## Conclusions

Critical Care Staff based RRS Team has an ability to diagnose and treat the patients at early phase of deterioration. MEWS at scene decreased at the end of 2013, which indicate the RRS allowed the early warning of the patients by the ward staffs. There is possibility that the system contributed to decrease UHD. We concluded that critical care staff based RRT is effective for patient safety and quality management in the hospital.

**Figure 1:**
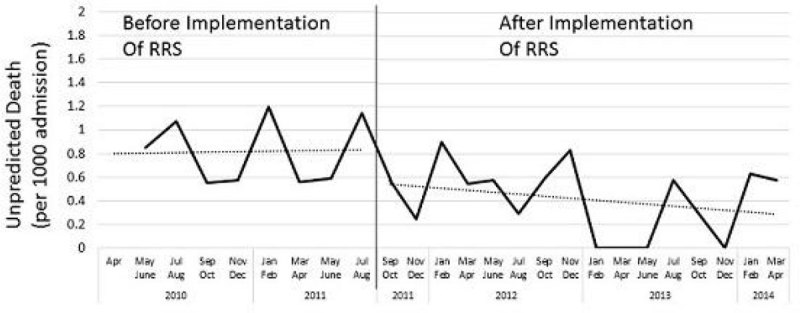
**The Change of Unpredicted Hospital Death.**

